# Prevention of Postoperative Graft Rupture by Additional Lateral Extra-articular Tenodesis in Anterior Cruciate Ligament Reconstruction: A Case Series of Four Patients

**DOI:** 10.7759/cureus.82790

**Published:** 2025-04-22

**Authors:** Hideaki Fukuda, Shigehiro Asai, Rei Yamaguchi, Keishin Ueno, Yamaura Ichiro, Hiroki Iwai

**Affiliations:** 1 Sports Medicine and Joint Center, Inanami Spine and Joint Hospital, Shinagawa, JPN; 2 Orthopedics, Hokusetsu Hospital, Takatsuki, JPN; 3 Orthopedics, Inanami Spine and Joint Hospital, Shinagawa, JPN; 4 Orthopedics, Funabashi Orthopedic Hospital, Funabashi, JPN; 5 Sports Medicine and Joint Center, Funabashi Orthopedic Hospital, Funabashi, JPN; 6 Spine Surgery, Iwai Orthopedic Medical Hospital, Edogawa, JPN

**Keywords:** anterior cruciate ligament reconstruction, case reports, iliotibial band, lateral extra-articular tenodesis, prevention

## Abstract

Anterior cruciate ligament (ACL) reconstruction is commonly performed; however, re-injury and graft failure remain significant concerns, particularly in high-demand individuals. Lateral extra-articular tenodesis (LET) has recently attracted attention as an adjunct to ACL reconstruction to reduce rotational instability and improve surgical outcomes. In this report, we describe four patients who underwent primary ACL reconstruction with additional LET. Despite experiencing significant traumatic knee events postoperatively, all four patients avoided ACL graft re-rupture. These cases suggest LET has a robust protective effect against ACL graft rupture, especially in patients at high risk for postoperative injury.

## Introduction

Anterior cruciate ligament (ACL) injuries frequently affect athletes and highly active individuals, with reconstruction surgeries widely performed to restore knee stability and function. Despite advancements, re-injury and graft ruptures remain prevalent concerns, particularly among younger, high-demand populations. Recent studies have indicated that lateral extra-articular tenodesis (LET), performed concurrently with ACL reconstruction, significantly enhances rotational knee stability and reduces ACL graft failure rates, especially in patients demonstrating risk factors such as high-grade pivot shift, generalized laxity, or involvement in pivoting sports.

Surgical indications for LET

Primary indications for performing LET include chronic ACL injuries, revision cases, cases exhibiting a high-grade pivot shift (Grade 2 or 3), or an increased posterior tibial slope greater than 12 degrees. Additional indications include participation in pivoting sports, generalized ligamentous laxity, knee hyperextension, and high-demand athletic activity.

Post-injury conservative management

After sustaining postoperative knee injuries, each patient underwent structured conservative management. Initially, management consisted of external immobilization for two weeks, partial weight-bearing, nonsteroidal anti-inflammatory drugs (NSAIDs), and icing to reduce inflammation. Subsequently, patients utilized hinged braces, previously used postoperatively, for four to six weeks. Range-of-motion exercises and muscle-strengthening training commenced immediately upon brace application. Patients without significant pain, swelling, or range-of-motion limitations began stationary cycling exercises at three weeks post-injury, followed by jogging between three to four weeks, contingent upon favorable clinical progress. Activities were gradually escalated, culminating in sport-specific activities. Muscle strength and functional recovery were assessed systematically after eight weeks to determine readiness for full return to sports.

## Case presentation

Case 1

**Figure 1:**
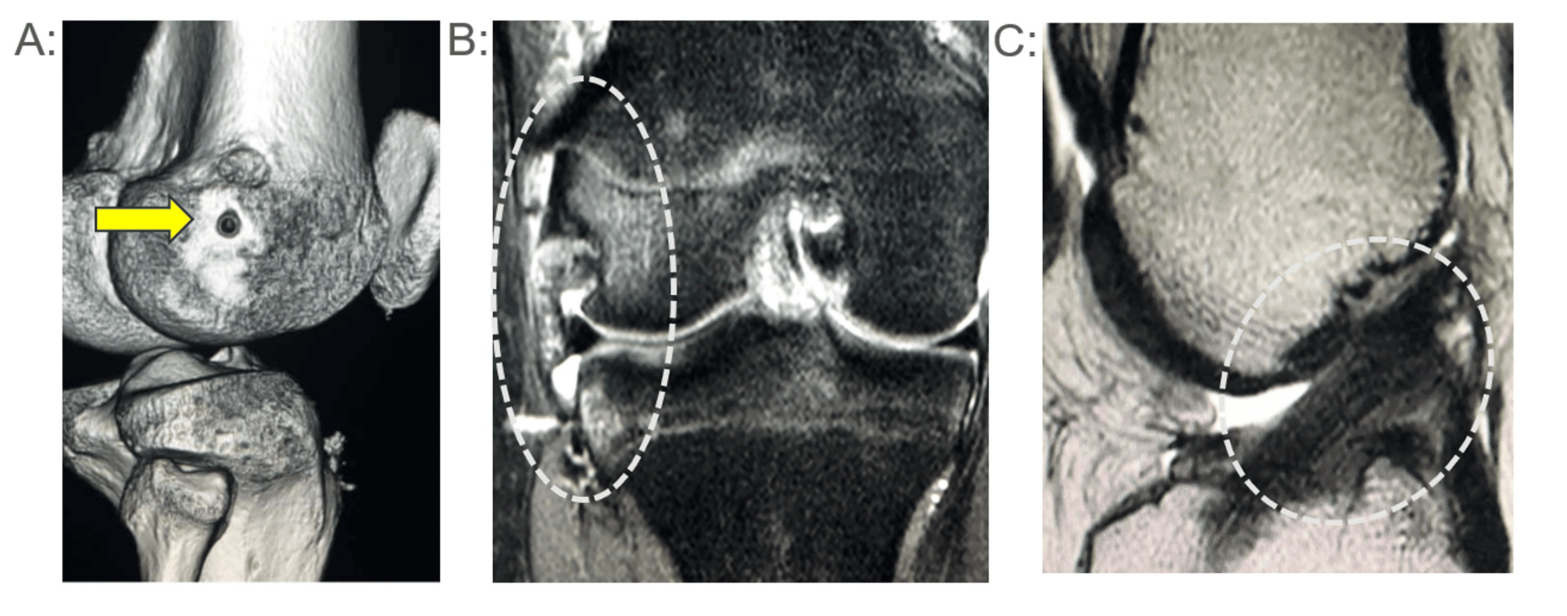
Case 1 (CT・MRI) (A) Postoperative CT scan. The arrow indicates the location of the bone tunnel for the lateral extra-articular tenodesis (LET). (B) MRI at the time of reinjury (fat-suppressed image): injury is observed at the site of the LET. (C) MRI at the time of reinjury (T2 sagittal image): the anterior cruciate ligament (ACL) is intact

A 16-year-old female national U16-level soccer player underwent primary ACL reconstruction using a bone-tendon-bone (BTB) autograft combined with LET augmentation. Thirteen months postoperatively, she sustained a noncontact knee injury. Postoperative computed tomography (CT) demonstrated an anatomically appropriate femoral tunnel location for LET. Magnetic resonance imaging (MRI) revealed injury localized at the LET site, while sagittal MRI confirmed intact ACL graft integrity. Following conservative therapy, she returned to competitive soccer within 2.2 months without ACL graft rupture (Figure 1).

Case 2

**Figure 2:**
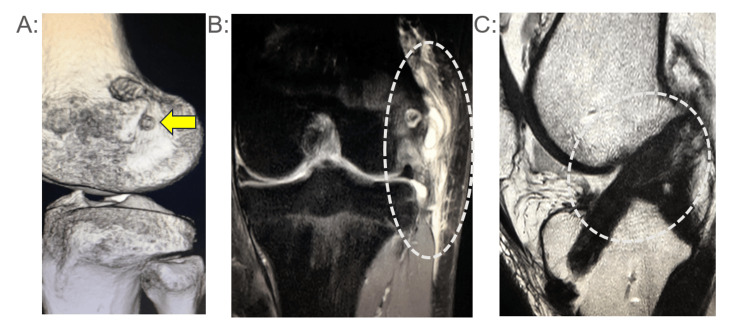
Case 2 (CT・MRI) (A) Postoperative CT image. The arrow marks the location of the bone tunnel created for the lateral extra-articular tenodesis (LET). (B) MRI at the time of reinjury (fat-suppressed sequence): evidence of damage is seen at the LET site. (C) MRI at the time of reinjury (T2-weighted sagittal view): the anterior cruciate ligament (ACL) remains intact

A 28-year-old male professional mixed martial arts (MMA) athlete underwent primary ACL reconstruction using a quadriceps tendon-bone (QTB) autograft with additional LET. Ten months post-surgery, he experienced a traumatic contact injury to his knee. Postoperative CT scans showed the correct anatomical placement of the femoral tunnel for LET. MRI evaluation revealed LET disruption, while sagittal T2-weighted images indicated that the ACL graft remained intact. After conservative management, he returned successfully to competitive activities within 2.4 months without graft failure (Figure 2).

Case 3

**Figure 3:**
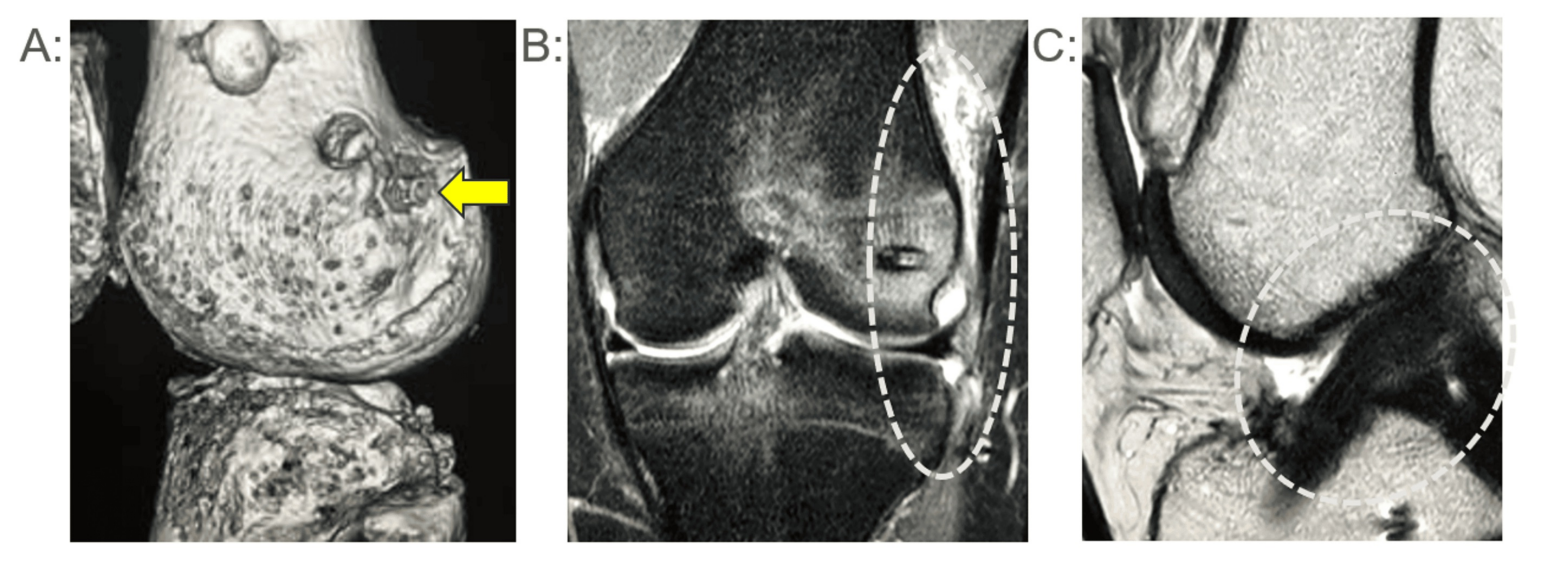
Case 3 (CT・MRI) (A) Postoperative computed tomography (CT) image illustrating the anatomical placement of the femoral tunnel for the lateral extra-articular tenodesis (LET), indicated by the arrow. (B) Magnetic resonance imaging (MRI) at the time of reinjury (fat-suppressed coronal sequence) reveals signal alteration and disruption at the LET construct site, suggestive of graft failure or structural compromise. (C) Sagittal T2-weighted MRI obtained at reinjury demonstrates preservation of anterior cruciate ligament (ACL) continuity, indicating no evidence of ACL rupture

An 18-year-old male basketball player (Tegner Activity Score 7) underwent ACL reconstruction using a BTB autograft augmented by LET. At 15 months post-surgery, he experienced a contact-related traumatic knee injury. CT imaging confirmed proper femoral tunnel placement for LET, whereas MRI (fat-suppressed coronal sequence) identified signal alteration and disruption indicative of LET structural compromise. Sagittal MRI demonstrated intact ACL continuity without rupture. Conservative management facilitated a successful return to sports within 2.0 months post-injury (Figure 3).

Case 4

**Figure 4:**
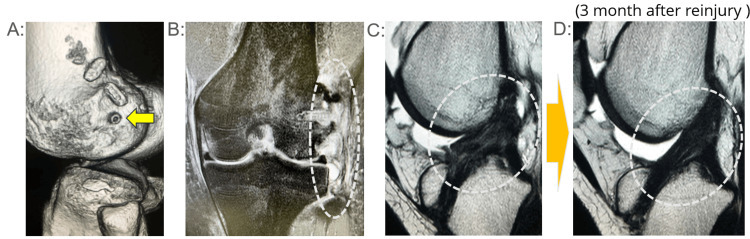
Case 4 (CT・MRI) (A) Postoperative computed tomography (CT) image showing the location of the femoral tunnel for the lateral extra-articular tenodesis (LET), indicated by the yellow arrow. (B) Magnetic resonance imaging (MRI) reveals extensive soft tissue damage extending from the region surrounding the tenodesis screw used for LET fixation to the entire iliotibial band (ITB). (C) The midsubstance of the reconstructed anterior cruciate ligament (ACL) demonstrates evident structural injury; however, partial fiber continuity is still preserved. (D) Follow-up MRI obtained three months after reinjury shows improved continuity of the ACL midsubstance, indicating spontaneous healing

A 17-year-old female basketball player (Tegner Activity Score 7) underwent primary ACL reconstruction utilizing a double-bundle hamstring autograft and LET augmentation. Eight months postoperatively, she sustained a contact injury during basketball play. Postoperative CT scans showed appropriate femoral tunnel placement for LET fixation. MRI examinations revealed extensive soft tissue injury involving the iliotibial band (ITB) and damage around the LET fixation site. Notably, the ACL midsubstance showed partial structural damage with preserved continuity. A follow-up MRI obtained three months post-injury demonstrated improved healing and continuity of the ACL graft. She returned to competitive basketball 3.6 months after conservative management (Figure 4).

## Discussion

ACL reconstruction remains a commonly performed surgical intervention, particularly among athletes and individuals who engage in high-demand pivoting sports. However, postoperative ACL graft ruptures and recurrent injuries remain significant clinical concerns, especially in young athletes returning to competitive sports. Several anatomical, biomechanical, and patient-related risk factors such as generalized ligamentous laxity, high-grade pivot shifts, increased tibial posterior slope, and participation in high-risk sports have been identified, potentially contributing to these re-injuries [1-5]. To address these challenges, LET has emerged as a valuable adjunctive procedure combined with ACL reconstruction [1-3,6-8]. The biomechanical rationale for LET is to augment rotational stability, thereby reducing excessive rotational forces acting on the ACL graft during high-demand activities [4-6,9,10].

Recent studies strongly support the notion that LET, performed in combination with ACL reconstruction, can significantly decrease the rate of postoperative ACL graft failure. Indeed, our presented case series of four patients who underwent primary ACL reconstruction with LET augmentation strongly supports the clinical benefit of this approach. Despite sustaining traumatic reinjuries postoperatively, all four patients successfully avoided ACL graft rupture, suggesting the protective effect of LET against potential graft failures during high-risk events. Current literature strongly supports our clinical experience, demonstrating improved outcomes when LET augmentation is incorporated into ACL reconstruction. For instance, Guy et al. demonstrated elite alpine skiers exhibited markedly lower rates of graft re-rupture (6.5%) when LET was performed in conjunction with ACL reconstruction, compared to isolated ACL reconstruction (34%) [3]. Similarly, Monaco et al. highlighted a significant difference in ACL graft rupture rates among adolescent athletes undergoing ACL reconstruction, with zero percent re-rupture rates in patients receiving LET versus a 15% rate in isolated ACL reconstruction [2]. Furthermore, Getgood et al. demonstrated significantly reduced re-rupture rates with combined LET and ACL reconstruction procedures (4%) compared to ACL reconstruction alone (11%) [1].

Additionally, systematic reviews and meta-analyses have shown that LET significantly improves postoperative rotational knee stability. Song et al. performed a meta-analysis of randomized controlled trials, concluding that the addition of LET to ACL reconstruction not only significantly reduces the graft rupture rate but also effectively improves anterior and rotational knee stability [5]. Recent publications by Boksh et al. [6] and Eggeling et al. [11] further emphasized LET's efficacy in reducing residual anterolateral instability and graft failure rates.

Despite these convincing data, some controversies remain concerning patient selection and universal indication for LET. Akoto et al. emphasized that routine augmentation might not be necessary for every ACL reconstruction patient, particularly those without evident rotational instability or high-grade pivot shifts [12]. Therefore, careful preoperative evaluation, individualized surgical indication, and patient-specific considerations remain critical factors when deciding whether LET augmentation should accompany ACL reconstruction.

## Conclusions

The presented four cases underscore the effectiveness of LET in primary ACL reconstructions, particularly for athletes and individuals at elevated risk for ACL re-injury. Adding LET may represent a valuable surgical strategy to significantly reduce the likelihood of ACL graft rupture following postoperative traumatic events.
